# Health-economic evaluation of statins versus lifestyle changes for cardiovascular disease prevention

**DOI:** 10.1371/journal.pone.0331176

**Published:** 2025-09-12

**Authors:** Afschin Gandjour

**Affiliations:** Frankfurt School of Finance & Management, Frankfurt, Germany; University of Magna Graecia: Universita degli Studi Magna Graecia di Catanzaro, ITALY

## Abstract

**Introduction:**

The German Federal Ministry of Health aims to improve cardiovascular health by expanding statin eligibility. However, the shift in funding from lifestyle-based preventive programs to statins has raised concerns that existing prevention efforts could be undermined. This study assessed the cost-effectiveness of statins compared with lifestyle interventions for the prevention of cardiovascular disease (CVD) in Germany under constrained healthcare budgets.

**Methods:**

A cost-effectiveness analysis was conducted using secondary data. Effectiveness data for statins were drawn from meta-analyses and systematic reviews, showing a 26% reduction in major cardiovascular events. Lifestyle intervention data were derived from the US Preventive Services Task Force (2020), reporting a relative risk of 0.80 for CVD events. A z-score test compared the effectiveness, while a Bayesian analysis assessed the probability of statins being more effective. Costs were analyzed from the perspective of German statutory health insurance (SHI) enrollees, including copayments and treatment monitoring expenses.

**Results:**

The z-score of −1.26 (p = 0.207) indicated no statistically significant difference in the effectiveness of statins compared to lifestyle interventions. However, Bayesian analysis estimated an 89.7% probability that statins are more effective. Statins were generally cheaper, especially when preventive courses are repeated annually.

**Conclusions:**

Statins likely offer higher cost-effectiveness than lifestyle changes, though the difference in benefits is not statistically significant. Concerns about reallocating funds from lifestyle programs to statins are somewhat mitigated by these findings.

## Introduction

The German Federal Ministry of Health (Bundesministerium für Gesundheit) recently introduced a draft bill titled *Entwurf eines Gesetzes zur Stärkung der Herzgesundheit (Gesundes Herz-Gesetz – GHG)* [1]. The primary objective of this draft legislation is to improve the early detection and treatment of cardiovascular diseases (CVDs), thereby enhancing heart health across the population through a series of targeted measures. CVD remains a leading cause of morbidity and mortality in Germany, imposing a significant economic burden on the healthcare system.

One key provision of this proposed law is the expanded eligibility for statin prescriptions for patients with known cardiovascular risk factors. This measure is expected to benefit an additional 2 million patients. Statins, widely used for lowering cholesterol levels, have been shown to significantly reduce the risk of myocardial infarctions (MIs) and strokes [2]. This expansion is projected to result in additional annual expenditures of around 90 million euros [1]. The proposed law stipulates that these costs should be accounted for within the expenditures that health insurance funds regularly allocate to health promotion and primary prevention services, as per German Social Code Book V (§ 20 paragraph 6).

The Association of Substitute Health Insurance Funds (Verband der Ersatzkassen e. V. – vdek), which represents the interests of six health insurance funds covering over 28 million people in Germany, has expressed concerns about this shift in financial priorities. Specifically, the vdek argues that expanding statin use shift resources toward the medicalization of health risks and undermines existing prevention efforts that emphasize healthier lifestyles and behavioral interventions. The association highlights that such a shift could weaken long-term preventive strategies by prioritizing pharmaceutical interventions over holistic health promotion, which they consider “completely outdated” [3]. This concern reflects a broader debate about balancing investments in proactive public health initiatives versus reactive medical treatments.

In Germany, approximately 1.5 million patients participate in *Präventionskurse* (preventive courses) as part of the broader prevention and rehabilitation framework. These courses are a fundamental component of the German health system, aimed at reducing the risk of chronic diseases and promoting long-term health through lifestyle changes. They typically address areas such as physical activity, nutrition, stress management, and addiction prevention. The goal of these programs is to educate individuals on how to achieve and maintain a healthy lifestyle, thereby preventing the onset of diseases and reducing the burden on the healthcare system. They are designed to be accessible, with costs often largely covered by statutory health insurance (SHI). Although evidence is limited, it suggests that these courses can improve subjective health assessments by encouraging sustainable lifestyle changes [4].

This study evaluates the trade-off between enrolling adult German patients in preventive health courses and treating them with statins, particularly in the context of limited healthcare resources. It seeks to determine which approach offers the most value for money in reducing cardiovascular disease risk and improving population health outcomes in the context of the proposed legislative changes. To achieve this, a cost-effectiveness analysis was conducted, comparing the use of statins versus preventive courses in reducing the incidence of cardiovascular events (strokes and MIs). The target population included individuals with known cardiovascular risk factors, both with and without prior cardiovascular disease. The analysis utilized secondary data.

## Methods

### Data inputs

This section provides an overview of the data sources used in the analysis, with further details presented in the subsections below. The analysis relied otn secondary data from published meta-analyses and systematic reviews. For statins, the relative risk reduction for cardiovascular events and associated costs were derived from Byrne et al. [2] and Dugré et al. [5]. For lifestyle interventions, effectiveness data were sourced from the US Preventive Services Task Force [6] review. Cost estimates for preventive courses were based on public information from German statutory insurers (e.g., [7,8]). Real-world adherence rates were obtained from a German claims database for statins [9] and systematic reviews for lifestyle interventions [10]. The analysis focused on cardiovascular outcomes (strokes and MIs) as primary endpoints.

### Effectiveness data

Given the limited availability of data on the effectiveness of German prevention courses—particularly on cardiovascular outcomes—this study utilized international randomized controlled trial (RCT) data as a proxy. Specifically, the effectiveness of lifestyle interventions on cardiovascular events was derived from a review by the US Preventive Services Task Force [6]. This review included English-language RCTs published up to July 2020 that examined the impact of behavioral counseling interventions aimed at improving diet and increasing physical activity.

The focus of the review was on primary prevention in adults with known CVD risk factors, such as hypertension, dyslipidemia, or metabolic syndrome, who had not yet experienced a cardiovascular event. The review assessed whether these primary care–relevant behavioral counseling interventions could improve CVD-related health outcomes, including morbidity and mortality. The analysis found that behavioral counseling was associated with a significant reduction in the risk of composite cardiovascular events, with a relative risk (RR) of 0.80 (95% CI, 0.73 to 0.87) [6]. The review was based on a search of MEDLINE, PubMed (publisher-supplied records only), PsycINFO, and the Cochrane Central Register of Controlled Trials, and was not restricted to studies conducted in the United States.

Statistically significant improvements were also observed in intermediate outcomes, including reductions in systolic blood pressure (–1.8 mm Hg), diastolic blood pressure (–1.2 mm Hg), total cholesterol (–3.5 mg/dL), low-density lipoprotein cholesterol (–2.1 mg/dL), fasting glucose (–2.3 mg/dL), weight (–1.6 kg), and body mass index (–0.5). However, the pooled effect on all-cause mortality did not show a statistically significant benefit (pooled RR, 0.89 [95% CI, 0.71 to 1.11]), likely due to the limited power of many studies to assess this outcome. Adverse events related to diet and physical activity counseling were exceedingly rare, with no statistically significant differences reported in any study for harms outcomes.

The review highlighted that no single optimal or representative intervention was identified, as a wide range of behavioral counseling approaches were found to improve health profiles. Programs demonstrating clear benefits generally offered group-based sessions ranging from 5 to 12 sessions over 4–12 months, or 20–30 sessions over 24 months if weight loss was a primary focus. The majority of trials recruited participants from healthcare settings, though most interventions did not involve a primary care clinician. Nearly all trials addressed dietary changes, with or without physical activity, and included at least 30 minutes of contact time. 

To ensure the most up-to-date information, a search was conducted in PubMed for reviews published between July 2020 and April 2025 that examined the impact of lifestyle interventions focused on diet and physical activity on cardiovascular outcomes. The following search algorithm was used: “(Cardiovascular[title] OR Heart[title]) AND (Disease[title] OR Diseases[title]) AND Prevent*[title] AND (Dietary OR ‘physical activity’ OR Exercise OR Nutrition) AND Review*”. To broaden the scope of the PubMed search, an alternative, more comprehensive algorithm was also applied: “((Cardiovascular[tiab] OR Heart[tiab]) AND (Disease[tiab] OR Diseases[tiab]) AND Prevent*[tiab]) AND (Dietary[tiab] OR “physical activity”[tiab] OR Exercise[tiab] OR Nutrition[tiab]) AND (“behavioral counseling”[tiab] OR “health counseling”[tiab] OR “lifestyle intervention”[tiab]) AND (Review[pt] OR systematic[sb])”. Additionally, a search was conducted in the Cochrane Library using the following algorithm: “(Cardiovascular OR Heart) AND (Dietary OR ‘physical activity’ OR Exercise OR Nutrition)”, with the review type restricted to Intervention Reviews.

To supplement these efforts, the study followed guidance from the University of Toronto Libraries [11] to search for grey literature using Google Advanced Search. The search terms included: ‘dietary,’ ‘physical activity,’ and ‘systematic review’ (all terms must be present) and ‘cardiovascular’ OR ‘heart’ (at least one of these terms must be present). Results were set to “all languages” to capture German and other non-English sources. As recommended, the first 100 results were reviewed.

Reviews focusing on specific interventions, such as the Mediterranean diet, or on specific populations (e.g., employees in workplace programs), were excluded, as they did not sufficiently represent the broad spectrum of interventions offered by German prevention courses. Similarly, reviews in patients without known CVD risk factors were excluded to align with the eligible patient population.

For statins, the effectiveness in reducing major cardiovascular events is well established. Based on the meta-analysis by Byrne et al. [2] covering primary and secondary prevention trials with comparators such as placebo, no treatment, or usual care, and an average follow-up of 4.4 years (range: 1.9–6.1 years), a combined relative risk reduction (RRR) for both stroke and MI was calculated. Using a weighted average approach that weighs each RRR by its respective absolute risk reduction (ARR), the combined RRR for stroke and MI was 25.5% (95% CI, [20.5%, 30.5%]). Statins also demonstrated a significant impact on all-cause mortality, with a relative risk of 0.91 (95% CI: 0.86–0.95). These findings are supported by a recent systematic review of RCTs showing a median reduction in major cardiovascular events by 26% [5]. It is important to note that most clinical trials report results based on an intention-to-treat (ITT) analysis, which includes all participants as originally allocated after randomization, regardless of adherence to the treatment regimen. Consequently, the reported RRR typically accounts for some level of non-adherence.

This paper presents a sensitivity analysis that adjusted RRs from RCTs of statins and lifestyle changes to account for real-world adherence, thereby estimating real-world RRs. For statins, adherence rates in the intervention arms of RCTs were approximately 70% at 5 years [12]. For behavioral counseling interventions, adherence rates in RCTs were estimated at 77% over 1–2 years [10], consistent with the relatively high adherence (>80%) typically observed in the first year of such interventions, followed by a decline over time. In terms of long-term discontinuation in real-world settings, a German claims data analysis of patients with dyslipidemia newly prescribed statins (n = 865,732) reported a persistence rate of 20.6% over 3 years [9]. For lifestyle modification programs focusing on diet and physical activity, long-term persistence rates are less well-documented, even internationally. Given the greater effort and motivational barriers associated with lifestyle programs, and the low-effort requirement of statins despite higher discontinuation rates due to side effects, the study assumed comparable 3-year adherence rates for both interventions. A best estimate for 3-year adherence to statins and lifestyle programs in real-world settings was set at 25%, reflecting the similar long-term challenges faced by both. Beyond the 3-year period, the study assumed that adherence rates for both statins and lifestyle programs would decline at the same rate. This assumption is conservative, as it may disadvantage statins, where adherence decline often slows after an initial drop as long-term adherers stabilize. In contrast, adherence to lifestyle programs may continue to decline at a faster pace due to higher effort and motivational barriers.

### Statistical analysis

#### Empirical methods justification.

The selection of empirical methods aimed to balance methodological rigor with transparency and parsimony. A direct comparison of relative risks using a z-test was chosen to test for statistical significance due to its simplicity and appropriateness when comparing independent estimates from different meta-analyses. A Bayesian analysis was conducted to estimate the probability that one intervention is more effective than the other, offering a more intuitive interpretation of the comparative effectiveness under uncertainty. Adjustments for real-world adherence were implemented to reflect effectiveness in routine care settings, given the known decline in adherence over time for both interventions.

#### Effectiveness comparison and adjustment for real-world adherence.

Given that meta-analyses of lifestyle interventions and statins reported a significant effect on CVD but not on other disease-specific outcomes, only CVD was used as the primary outcome in this study. The Bucher indirect treatment comparison (ITC) method was not used, as it requires interventions being compared to share a common baseline comparator, typically placebo or standard care. In studies comparing statins to placebo, participants received basic lifestyle advice, such as brief written or verbal guidance on healthy eating and exercise, but not an intensive intervention. The comparators in the US Preventive Services Task Force [6] review of behavioral counseling interventions were not explicitly detailed. It is inferred that the comparator for the behavioral counseling interventions likely involved no intervention, standard care, or a minimal intervention not including the specific components of intensive behavioral counseling being evaluated. This potential discrepancy in comparators could violate the common comparator assumption required for a Bucher ITC.

To determine whether there is a statistically significant difference between the two interventions (statins versus behavioral counseling), a z-score was calculated for the difference between their relative risks (RRs). The z-score was then used to compute the p-value. The formula for the z-score is as follows:


$$z=\frac{\text{log}\left({RR}_{1}\right)-\text{log}\left({RR}_{2}\right)}{\sqrt{{SE}_{1}^{2}+{SE}_{2}^{2}}},$$ (1)

where ${RR}_{1}$ and ${RR}_{2}$ are the relative risks for statins and behavioral counseling, respectively, and ${SE}_{1}$ and ${SE}_{2}$ are the standard errors of the log-transformed relative risks. The p-value is derived from the z-score using the cumulative distribution function (CDF) of the standard normal distribution:


p=2×[1−Φ(|z|)]. (2)

In addition to the frequentist approach, a Bayesian calculation was performed to determine the probability that statins are more effective than lifestyle changes, using uninformative priors. The process involved the following steps:

1. Uninformative normal priors were assumed for the log relative risks (log-RRs) of statins and lifestyle changes, with a mean of 0 and a large variance (σ^2^ = 1000^2^).
2. The observed log-RRs and their standard errors were used as the likelihood in the Bayesian model.
3. The posterior mean for the log-RR of statins and lifestyle changes was calculated using the formula:

$$\text{Posterior}\;{\text{Mean}}_{\text{statins}}=\frac{\frac{\text{log}{RR}_{\text{statins}}}{{SE}_{\text{statins}}^{2}}}{\frac{1}{\sigma_{\text{prior}}^{2}}+\frac{1}{{SE}_{\text{statins}}^{2}}},$$ (3)

$$\text{Posterior}\;{\text{Mean}}_{\text{lifestyle}}=\frac{\frac{\text{log}{RR}_{\text{lifestyle}}}{{SE}_{\text{lifestyle}}^{2}}}{\frac{1}{\sigma_{\text{prior}}^{2}}+\frac{1}{{SE}_{\text{lifestyle}}^{2}}}.$$ (4)

1. The posterior variance for each log-RR was calculated using:

$${\text{Posterior}\;\text{Variance}}_{\text{statins}}=\frac{1}{\frac{1}{\sigma_{\text{prior}}^{2}}+\frac{1}{{SE}_{\text{statins}}^{2}}},$$ (5)

$${\text{Posterior}\;\text{Variance}}_{\text{lifestyle}}=\frac{1}{\frac{1}{\sigma_{\text{prior}}^{2}}+\frac{1}{{SE}_{\text{lifestyle}}^{2}}}.$$ (6)

1. The difference in posterior means between statins and lifestyle changes was calculated, along with the combined standard error of this difference. The probability that statins are more effective than lifestyle changes was then determined using the CDF of the standard normal distribution:

$$P(\text{Statins}\;\text{more}\;\text{effective})=\Phi \left(\frac{\text{Diff}\;\text{Mean}}{{SE}_{\text{combined}\;\text{posterior}}}\right).$$ (7)

This Bayesian approach thus combines prior beliefs (uninformative in this case) with observed data to produce a posterior distribution, from which the probability of statins being more effective than lifestyle changes is derived. This method allows for a more nuanced interpretation of the data, taking into account both the observed evidence and prior uncertainty.

In the sensitivity analysis, RRs from RCTs of statins and lifestyle changes were adjusted for real-world adherence to estimate real-world RRs. These adjusted RRs represent the average impact of statins and lifestyle changes over a specific time frame and were subsequently used to calculate the difference in posterior means between statins and lifestyle changes. Due to the differential impact of adherence on clinical outcomes, different adjustment approaches were applied for statins and lifestyle changes.

For statins, which require continuous use to maintain their pharmacological effect, a linear relationship between adherence rate and RRRs [13–15] as well as between duration of adherence and RRRs (based on Fig 2 in [16]) was assumed:


$${RR}_{\text{real}\;\text{world}}=1-\left(\frac{1-{RR}_{\text{RCT}}}{A_{\text{RCT},\text{avg}}}\right){\times A}_{\text{real}\;\text{world},\text{avg}},$$ (8)

where $A_{\text{avg}}$ refers to the average adherence over the study period. To account for both the linear decline in adherence and the effect of discounting, the average adherence over the study period was calculated using the following equations:


$$A_{\text{avg}}=\frac{\int_{0}^{T}A(t)\cdot e^{-rt}dt}{\int_{0}^{T}e^{-rt}dt},$$ (9)

$$A(t)=A_{0}-\frac{A_{0}-A_{T}}{T}\cdot t,$$ (10)

where T denotes the total time frame, $e^{-rt}$ is the discount factor, and r is the discount rate. At time 0, patients exhibit perfect adherence ($A_{\text{0}}=1)$, which linearly declines over time until T. Maintaining a higher real-world adherence over time increases the average real-world adherence (per Equation 10), which in turn yields a lower RR in the real world (per Equation 8), indicating higher effectiveness.

For lifestyle interventions, we mapped adherence to effect with a concave exponential function to capture diminishing returns [17,18], so that early adherence yields larger gains and even partial adherence delivers a substantial share of the maximal benefit:


$${RR}_{\text{real}\;\text{world}}={{RR}_{\text{RCT}}}^{A_{\text{real}\;\text{world},\text{avg}}/A_{\text{RCT},\text{avg}}}.$$ (11)

The average adherence over the study period was calculated according to Equations 9 and 10. The ratio $A_{\text{real}\;\text{world}}/A_{\text{RCT}}$ in the exponent is unaffected by the relationship between the duration of adherence to lifestyle interventions and RRs, assuming that the effect of duration impacts both real-world and RCT settings similarly.

These formulas (Equations 8–11) were applied to the means of the trial-based RRs as well as to the limits of their 95% confidence intervals.

Separating adherers and non-adherers helped account for the disutility associated with statin use and participation in lifestyle programs. The disutility from taking a statin pill every day was assumed to be small (0.001) in one study [19]. On the other hand, participating in lifestyle programs is also likely to involve some disutility. Participants must dedicate time to attend classes, workshops, or activities, which may interfere with personal or professional responsibilities. Stress or frustration could arise if participants perceive the program as too demanding or if progress is slower than expected.

Adherers to statins are less likely to experience severe side effects, so the disutility for adherers is expected to be even smaller than 0.001. Similarly, adherers to lifestyle programs likely experience less disutility than dropouts, as they are more likely to find the program manageable, experience positive outcomes, and successfully integrate the activities into their lives. Given the lack of empirical data, we therefore assumed that the disutility for adherers to both statins and lifestyle programs is negligible.

Dropouts, on the other hand, often discontinue participation due to higher perceived burdens or dissatisfaction, which reflects greater disutility during their participation. However, treatment discontinuation, irrespective of cause, was modeled in the sensitivity analysis through adjustments for real-world adherence, as described above. While patients experiencing adverse events may suffer disutility during their period of adherence, this disutility is time-limited and ends once they discontinue the intervention.

Given the lack of empirical data on the disutility experienced by non-adherers, it may be reasonable to assume that the disutility for non-adherers to statins and lifestyle programs during their shortened period of adherence effectively cancels out.

### Cost-effectiveness

The analysis was conducted from the perspective of SHI enrollees. Unlike the perspective of the sickness funds themselves, this viewpoint includes copayments made by the enrollees, allowing for a more comprehensive analysis. It also enables the determination of the level of copayments that would make both treatment options—statins and lifestyle interventions—economically equivalent.

For the cost of statins, the analysis considered the pharmacy retail price after legally mandated discounts from the reference price. Atorvastatin was selected as the representative statin, given its frequent use in the prevention of cardiovascular diseases [20]. The commonly recommended dose for adults, particularly for the prevention of cardiovascular events, is 40 mg. In a sensitivity analysis, the cheapest statin dose was also considered.

The annual total costs for the treatment and monitoring of a patient taking statins for the prevention of cardiovascular diseases range from €31 to €62, excluding medication costs [21]. These costs include regular doctor visits for general and extended examinations (EBM codes 03220 and 03230 [22]), which cost approximately €15 per visit and occur 1–2 times per year. Laboratory tests, such as lipid profiles (EBM codes 32060, 32061, 32062, 32063), cost €0.15 per test and are performed 1–2 times per year. Liver function tests (EBM code 32070) cost €0.25 per test and are conducted as needed. Additional specific tests, such as creatine kinase determination (EBM code 32074), can cost €0.25 per test and are also required as needed ( [23], p. 162).

It is estimated that 12% of statin users in Germany require additional clinical visits annually due to side effects [20]. The cost per patient for these additional visits ranges from €19 to €60, depending on the type and severity of the side effects. These visits are often due to issues such as severe muscle pain, new-onset diabetes, significant liver enzyme elevation, and severe gastrointestinal problems.

The course fees for group-based behavioral counseling interventions designed to improve diet, increase physical activity, and reduce sedentary behavior typically cover 5–12 sessions over 4–12 months. These fees vary based on several factors, including the specific course provider, the type of course, and the geographical location. The total cost for such preventive courses generally ranges from €100 to €300, depending on the program’s length and intensity [7]. The out-of-pocket costs for these courses, after considering typical reimbursements provided by major German health insurance companies, generally range from €20 to €150 [8].

For both interventions, continuous application was assumed in the base case to achieve the required health benefits. For lifestyle programs, the base-case analysis assumed that preventive courses would be repeated annually. The systematic review by the US Preventive Services Task Force [6] included trials that assessed cardiovascular events over periods ranging from 1 to 16 years. Interventions typically lasted 6–18 months; in the base case, we assumed the total follow-up (study duration) did not extend meaningfully beyond the intervention period. In a sensitivity analysis, it was assumed that preventive courses would not need to be repeated annually but rather would be required several times over a 10-year period (e.g., every 2–3 years). Preventive courses aim at promoting long-term lifestyle changes and are often designed to equip individuals with the tools and knowledge to maintain these changes over an extended period [24]. The intent is that once completed, participants can continue to apply the principles learned without needing to repeat the course frequently [24].

Since cardiovascular events were the primary health outcome of interest, rather than metrics such as quality-adjusted life years, the study did not specify a fixed time horizon in the base case. For the sensitivity analysis, which accounted for real-world adherence and the increasing effectiveness of statins with longer adherence, a 3-year time horizon was used due to the limited availability of longer-term real-world adherence data. An annual discount rate of 3% was applied to both benefits and costs over the time horizon. All costs used in the analysis were adjusted to 2024 euros.

## Results

### Literature search

A PubMed search conducted on April 25, 2025, yielded 247 results. Except for one, all hits failed to meet the inclusion criteria outlined in the “Effectiveness data” section. An umbrella review of systematic reviews on interventions for the primary prevention of cardiovascular disease, published up until March 2021, was identified [25]. However, the review by the US Preventive Services Task Force [6] was not included in this umbrella review. One systematic review included in the umbrella review examined multicomponent interventions, with the last searches conducted in June 2014 and published in 2015. These interventions included various types of nutritional supplements, along with diet and physical activity advice. The AMSTAR rating for this review was 15 out of 16. The combination of dietary interventions and physical activities showed little or no significant beneficial effect on major cardiovascular events. This review was excluded because its last search date was six years prior to that of the US Preventive Services Task Force review, which included more recent literature.

A search in the Cochrane Library conducted on April 25, 2025, yielded 182 Cochrane reviews, none of which met the inclusion criteria. Similarly, the grey literature search did not retrieve any additional relevant reviews. Based on these findings, the current study did not incorporate additional evidence from reviews beyond the publication by the US Preventive Services Task Force [6].

### Difference in effectiveness

Table 1 presents the relative risks associated with statins and behavioral counseling. To determine if the difference in effectiveness between statins and behavioral counseling was statistically significant, a z-score of −1.26 and a p-value of 0.207 were calculated. The p-value is greater than the conventional significance level of 0.05, indicating that the difference in effectiveness between statins and behavioral counseling is not statistically significant at an alpha level of 0.05. Therefore, the null hypothesis that the two interventions have the same effect cannot be rejected. The overlap in 95% confidence intervals, supported by the p-value, suggests that any observed difference could be due to chance.

**Table 1:** Base-case results based on evidence from randomized controlled trials (means and ranges). The comparator is no intervention.

Intervention	Effectiveness (relative risk)	Annual costs (€)
Statins	0.745 (95% CI, 0.695–0.795)	120.22 (range: 78.18–138.60)
Behavioral counseling	0.8 (95% CI, 0.73–0.87)	200 (range: 100–300)

CI, confidence interval.

A Bayesian calculation estimated the probability that statins are more effective than lifestyle changes to be 89.7%. This calculation indicates that, given the observed data, there is an 89.7% probability that statins are more effective than lifestyle changes in reducing cardiovascular events. This result aligns with the finding that the relative risk for statins is lower than that for lifestyle changes, indicating greater effectiveness. The posterior probability suggests that statins are likely superior to lifestyle changes based on the observed data and the uninformative priors used in the Bayesian analysis.

After adjusting for non-adherence in the real world, the RRs for both statins and lifestyle programs increased, reflecting a reduction in effectiveness (Table 2). Using Equations 8 and 11, the real-world RRs were calculated as 0.81 for statins and 0.84 for lifestyle programs (Table 2). Although the p-value of 0.33 exceeds the typical significance threshold (e.g., 0.05), the Bayesian analysis estimated an 83% probability that statins are more effective than lifestyle programs.

**Table 2:** Sensitivity analysis results based on real-world adherence (means and ranges). The comparator is no intervention.

Intervention	Effectiveness (relative risk)	Annual costs (€)
Statins	0.81 (95% CI, 0.77–0.84)	120.22 (range: 78.18–138.60)
Behavioral counseling	0.84 (95% CI, 0.78–0.90)	200 (range: 100–300)

CI, confidence interval.

### Cost-effectiveness

The annual treatment costs of statins, including monitoring and managing side effects, were estimated to average €120.22. If preventive courses were repeated annually, they would be more expensive than statins. However, if the preventive course is required only a few times over a 10-year period (e.g., every 2–3 years), the average annual cost could potentially be lower than that of statins (Table 3).

**Table 3:** Sensitivity analysis results based on biennial preventive courses (means and ranges). The comparator is no intervention.

Intervention	Effectiveness (relative risk)	Annual costs (€)
Statins	0.81 (95% CI, 0.77–0.84)	120.22 (range: 78.18–138.60)
Behavioral counseling	0.84 (95% CI, 0.78–0.90)	100 (range: 50–150)

CI, confidence interval.

## Discussion

The results indicate that statins have a higher probability of being more effective than lifestyle interventions and are unlikely to be more expensive. However, the base-case difference in benefits is not statistically significant due to the considerable overlap in the 95% confidence intervals. Despite this, Bayesian analysis suggests approximately a 90% probability that statins are more cost-effective than lifestyle interventions. When accounting for real-world adherence to statins and lifestyle interventions, this probability decreases slightly to 83%. Further supporting the analysis, statins demonstrated a significant impact on all-cause mortality, with a relative risk of 0.91 [2]. In contrast, behavioral counseling interventions did not show a significant effect [6]. However, behavioral counseling interventions showed significant effects on several intermediate endpoints, the additional utility of which beyond CVD remains unclear. Nevertheless, extrapolating intermediate endpoints when results from final endpoints are available may introduce unnecessary uncertainty or bias.

A previous cost-effectiveness analysis [26] comparing statins with lifestyle interventions in young adults from a US healthcare sector perspective also found that lifestyle interventions were more costly and less effective than statin therapy. Unlike the current analysis, which uses direct evidence on hard endpoints, this study projected reductions in coronary heart disease and stroke based on LDL-C reduction as an intermediate endpoint.

Overall, these findings suggest that reallocating resources from lifestyle programs to statins might improve patient outcomes without incurring additional costs, potentially easing concerns among sickness funds. Nonetheless, vdek’s [3] reservations about undermining prevention efforts should be carefully considered. While the results highlight the benefits of statins, the broader societal and health impacts of lifestyle interventions—particularly their potential long-term contributions to reducing the burden of non-communicable diseases—are not fully addressed in this analysis.

Additional limitations of the analysis must be acknowledged. The analysis relied on international data as a proxy for the effectiveness of German preventive courses, due to the lack of long-term evaluations specific to Germany. This reliance on international data may limit the relevance of the findings to the German healthcare context, as the design and implementation of preventive courses can vary significantly between countries. For example, unlike the interventions analyzed by the US Preventive Services Task Force [6], which often include one-on-one sessions with an interventionist and incorporate motivational interviewing, these elements are typically absent in German preventive courses, potentially reducing their expected benefits. Therefore, the use of international data—which reflects generally more intensive interventions than those typically available in Germany—likely reinforces the conclusions of this study.

A key strength of this study was the use of a parsimonious model to determine cost-effectiveness. Parsimony is a foundational principle in science, emphasizing that models and explanations should be as simple as possible while adequately accounting for observed phenomena. Parsimonious models enhance clarity, improve efficiency, and facilitate understanding, often requiring fewer resources to generate meaningful insights. This principle is widely recognized as essential for developing robust scientific theories [27].

Parsimonious modeling is also highly valued in health-economic decision modeling, where simpler models can reduce computational burden, enhance transparency, and improve interpretability, making the results more actionable for policymakers [28,29]. Factors that increase policymakers’ confidence in models include ease of understanding, clear presentation of assumptions and limitations, intuitive results, and transparency [30].

Including downstream cost savings from preventing MIs and strokes would not alter the probability of cost-effectiveness in this study, as the latter is primarily determined by the probability of effectiveness. This finding is consistent with earlier research by Gandjour [31].

Nevertheless, the absence of direct head-to-head trials comparing statins and lifestyle interventions specifically within the German population represents a critical gap. Such trials would offer more robust evidence on the relative effectiveness of these interventions and their impact on health outcomes and healthcare costs in the German context.

Future research should prioritize conducting direct head-to-head trials that compare the effectiveness of statins and lifestyle interventions in the German population, evaluating both short-term and long-term outcomes, and considering factors such as adherence rates and patient preferences. There is also a need for more research focused on the long-term effectiveness of German preventive courses specifically. This would clarify whether the international data used in this study accurately reflects the potential benefits of these courses within Germany.

Future research could also explore the potential benefits of stratifying patients based on their cardiovascular risk profiles when deciding between statins and lifestyle interventions. Additionally, investigating personalized approaches that combine pharmacological and lifestyle interventions could optimize outcomes for different patient populations [32]. Addressing these areas in future research would provide more definitive guidance on the most effective strategies for preventing cardiovascular disease in Germany.

## Conclusion

This study evaluated the cost-effectiveness of statins versus lifestyle interventions for the prevention of cardiovascular disease in Germany. The findings suggest that while the difference in effectiveness between the two interventions is not statistically significant, statins are likely to offer greater value for money. Given the probability of statins being more effective and less costly in most scenarios, reallocating preventive resources toward statins may be justified from an economic perspective. However, the broader societal and long-term health benefits of lifestyle interventions—particularly in promoting holistic health—should not be overlooked. Future research should explore combined strategies and generate better real-world evidence for German prevention programs.
